# A Comprehensive Meta-Analytical Investigation into the Incidence of Neonatal Amino Acid Metabolic Disorders Across China

**DOI:** 10.3390/ijns12030061

**Published:** 2026-07-30

**Authors:** Qiongfang Yao, Shuting Huang, Fei Kong, Min Wu, Xiaolong Qiu, Peiran Zhao, Yinglin Zeng, Jinying Luo, Jinfu Zhou, Liangpu Xu

**Affiliations:** 1 Fujian Maternity and Child Hospital College of Clinical Medicine for Obstetrics & Gynecology and Pediatrics, Fujian Medical University, Fuzhou 350001, China; yaoqiongfang00703@fjmu.edu.cn (Q.Y.); hst18350320353@fjmu.edu.cn (S.H.); kongfei@fjmu.edu.cn (F.K.); wm2002@fjmu.edu.cn (M.W.); 15394536832@163.com (X.Q.); zhaopeiran3@163.com (P.Z.); zengyinglin@fjsfy.com (Y.Z.); 2 School of Medical Technology and Engineering, Fujian Medical University, Fuzhou 350122, China; 3 Department of Preventive Medicine, School of Public Health, Fujian Medical University, Fuzhou 350122, China

**Keywords:** amino acid metabolic disorders, incidence, meta-analysis, newborn screening, China, regional variation

## Abstract

Amino acid metabolic disorders (AAMs) are a group of inherited metabolic diseases caused by defects in enzymes or transporters involved in amino acid metabolism. This systematic review and meta-analysis aimed to evaluate the incidence, disease spectrum, and regional distribution of AAMs in China. A comprehensive search of PubMed, Embase, Web of Science, and major Chinese databases identified studies published between January 2002 and December 2025. After rigorous screening and quality assessment, 65 studies were included, encompassing 16,757,850 newborns and 2928 confirmed AAM cases. The most prevalent subtypes included hyperphenylalaninemia (HPA), hypermethioninemia (MET), citrin deficiency (CD), citrullinemia type 1 (CTLN1), maple syrup urine disease (MSUD), ornithine transcarbamylase deficiency (OTCD), and tyrosinemia (HT). The pooled incidence of AAMs was estimated at 184.0 (95% confidence interval 155.0–218.0) per million newborns. Significant regional differences were observed in the overall incidence of AAMs, with a higher incidence in northern China than southern China (287.0 vs. 126.0 per million, *p* < 0.0001). This difference was largely attributable to the substantially higher prevalence of HPA in northern China, whereas other major AAM subtypes showed no significant north–south differences. In contrast, no significant north–south differences were identified for other major subtypes. Additionally, the proportion of tetrahydrobiopterin deficiency (BH4D) among HPA cases was significantly higher in southern China (*p* < 0.001). These findings provide comprehensive epidemiological evidence on AAMs in China and highlight the importance of region-specific newborn screening strategies.

## 1. Introduction

Amino acid metabolism disorders (AAMs) are a group of inherited metabolic diseases caused by congenital defects of key enzymes or transporter proteins involved in the synthesis, catabolism, or transport of amino acids, most of which follow an autosomal recessive inheritance pattern [1]. Blockage of the metabolic pathways results in the accumulation of specific amino acids and their toxic metabolites in the body, along with insufficient production of downstream products, which in turn causes multi-system involvement dominated by central nervous system damage, with clinical manifestations including intellectual developmental disorder, growth retardation, epileptic seizures, and, in severe cases, acute metabolic decompensation and even death [2]. Affected infants often lack specific manifestations during the neonatal period, presenting only with non-specific symptoms such as feeding difficulties, lethargy, or vomiting, making them susceptible to missed diagnosis or misdiagnosis. Therefore, early diagnosis through newborn screening (NBS) and timely intervention are of great significance for preventing irreversible neurological damage and improving the prognosis of affected children.

NBS for inherited metabolic diseases is a critical public health strategy that enables early diagnosis of inherited metabolic diseases and significantly improves prognosis [3,4]. Tandem mass spectrometry (MS/MS) has become the core technical approach for current NBS owing to its advantages of high sensitivity, high specificity, and the requirement for only a trace amount of dried blood spot sample [5], and is widely applied in the detection of multiple categories of inherited metabolic diseases, including organic acidemias, AAMs, fatty acid oxidation disorders, and urea cycle disorders [6].

China gradually introduced MS/MS for expanded NBS in the early 2000s, and it has now become a core technical tool for screening various AAMs, including hyperphenylalaninemia, maple syrup urine disease, and citrin deficiency [7]. Multicenter studies and systematic epidemiological analyses have shown regional variations in the overall screening detection rate of inherited metabolic diseases in China, with an overall incidence ranging from approximately 1/2000 to 1/7000, among which amino acid metabolic disorders represent one of the major categories [8,9,10,11]. It is of great clinical and public health significance to determine the overall incidence of AAMs in the Chinese population and regional incidence differences based on the Qinling–Huaihe Line as the geographical boundary between northern and southern China. Systematic differences in natural environment, geographical characteristics, population genetic background, and lifestyle brought about by the Qinling–Huaihe Line may influence the incidence of different subtypes of these diseases. At present, systematic evidence-based data at the national level remain scarce regarding north–south incidence disparities for some rare subtypes of AAMs. Clarifying the overall incidence, subtype distribution, and regional epidemiological characteristics of AAMs is crucial for guiding the optimization of newborn disease screening policies, as well as the overall planning and precise allocation of medical resources in China.

Therefore, the present meta-analysis systematically integrates high-quality epidemiological study data published domestically in China to comprehensively elucidate the overall epidemiological characteristics and national incidence level of AAMs in the Chinese population. We further analyze the regional incidence disparities based on the Qinling–Huaihe Line, the geographical boundary between northern and southern China, clarify the differences in epidemiological patterns and disease spectrum composition of AAMs between the northern and southern regions, and provide an evidence-based foundation for the optimization of neonatal disease screening strategies and the establishment of a regional prevention and control system in China.

## 2. Materials and Methods

### 2.1. Literature Search

This systematic review and meta-analysis was conducted in accordance with the PRISMA reporting guidelines, and the study protocol has been registered in PROSPERO (Registration ID: CRD420251120094; https://www.crd.york.ac.uk/PROSPERO/view/CRD420251120094; accessed on 27 October 2025) [12]. A systematic literature search was performed by three independent investigators (ZJF, LJY, and XLP), aiming to identify observational studies regarding NBS for AAMs published from January 2002 to December 2025. The search strategy covered English databases including PubMed, Embase, and Web of Science, as well as the China National Knowledge Infrastructure, Veipu, and Wanfang. The detailed search terms are as follows: (“amino acid disorders” OR “amino acid metabolic disorders” OR “inborn errors of metabolism”) AND (“newborn screening”) AND (“China” OR “Chinese”). In addition to the database searches, manual searching of the reference lists of the included studies was conducted as a supplementary measure to comprehensively capture all relevant literature.

### 2.2. Eligibility and Exclusion Criteria

Studies were eligible for inclusion if they met the following criteria: (1) Original observational studies conducted in the Chinese population; (2) Studies that reported the incidence or screening data of AAMs in newborns from different provinces and municipalities in China; (3) Studies with a study period spanning from 2002 to 2025; (4) Studies with moderate or higher methodological quality (AHRQ score ≥ 4). Studies that failed to meet the above criteria were excluded.

In addition, studies were excluded if they met any of the following conditions: (1) Duplicate publications; (2) Studies with overlapping screening populations; (3) Studies that only reported a single AAM subtype and from which the total number of all AAM cases and corresponding overall incidence could not be extracted (to ensure homogeneity of screening panels across included studies); (4) Studies whose subjects were hospitalized newborns or high-risk screening populations; (5) Literature not published in Chinese or English.

Specifically, for the identification and exclusion of overlapping cohorts, we jointly verified four core dimensions: geographic coverage, screening time window, data source, and author affiliation. Notably, criterion (3) does not systematically exclude all single-disorder studies. It applies only to studies from which the overall AAM incidence cannot be derived, to ensure consistent full-spectrum AAM screening panels across all included studies. This design reduces heterogeneity from variable screening scope and prevents systematic underestimation of overall AAM burden. The potential selection bias associated with this criterion has been fully addressed in the Limitations section.

### 2.3. Data Extraction

Data were independently extracted into a pre-designed data extraction form by two investigators (HST and YQF). The information extracted from the included primary studies included: first author, publication year, study time span, geographical region (province/municipality), number of screened newborns, types of AAMs covered by the screening, and the number of confirmed cases of AAMs. The estimated incidence of AAMs was calculated based on the extracted data. Any discrepancies between the two investigators during the extraction process were resolved through discussion and consultation with a third senior investigator (XLP) of the research team. As all data used in this meta-analysis were derived from publicly published peer-reviewed literature and did not involve direct intervention in human subjects, no additional ethical approval or patient informed consent was required.

### 2.4. Quality Assessment

The observational studies included in this meta-analysis were assessed in accordance with the standards of the Agency for Healthcare Research and Quality (AHRQ). This assessment process was independently completed by two investigators (HST and YQF). The detailed scoring criteria were as follows: 1 point was awarded for full compliance with each criterion, and 0 points were assigned for non-compliance or unclear reporting. The total score (ranging from 0 to 11 points) was calculated accordingly, and the methodological quality of the studies was classified into three grades based on the total score: high (8–11 points), moderate (4–7 points), and low (0–3 points). Any discrepancies arising from the initial assessment were resolved by the involvement of a third investigator (XLP), who conducted the deliberation and made the final adjudication (Attachment S1).

### 2.5. Statistical Analyses

All statistical analyses were performed using the meta and metafor packages in R version 4.5.3 (R Foundation for Statistical Computing, Vienna, Austria). Incidence was used as the primary effect size, and the Freeman–Tukey double arcsine transformation was applied for variance stabilization. After pooling, the results were transformed back to the original proportion scale along with their 95% confidence intervals (CIs).

Given the anticipated substantial between-study heterogeneity derived from differences in study design and population characteristics, a random-effects model was adopted as the primary analytical framework for all pooled estimates, as it accounts for both within-study sampling error and between-study variance and is methodologically appropriate for heterogeneous study populations. Between-study heterogeneity was assessed using Cochran’s Q test and the *I*^2^ statistic.

Prespecified subgroup analyses stratified by geographical region (southern and northern China) were performed under the random-effects framework, and a mixed-effects model was used to test for statistical differences between subgroups. The robustness of the pooled estimates was evaluated via leave-one-out sensitivity analysis, in which each study was sequentially omitted to examine its influence on the overall results and verify the stability of the findings.

For disease subtypes with ≥10 included studies, publication bias was assessed visually by funnel plots and quantitatively by Egger’s regression test and Begg’s rank correlation test. The trim-and-fill method was further applied to adjust for potential publication bias and estimate the impact of missing studies on pooled estimates. A *p*-value < 0.10 was considered indicative of potential publication bias. Notably, for rare subtypes with extremely low event counts, the statistical power of these tests is limited; thus, the results are presented for exploratory reference only and not as definitive evidence of publication bias.

## 3. Results

### 3.1. Study Selection

**Figure 1:**
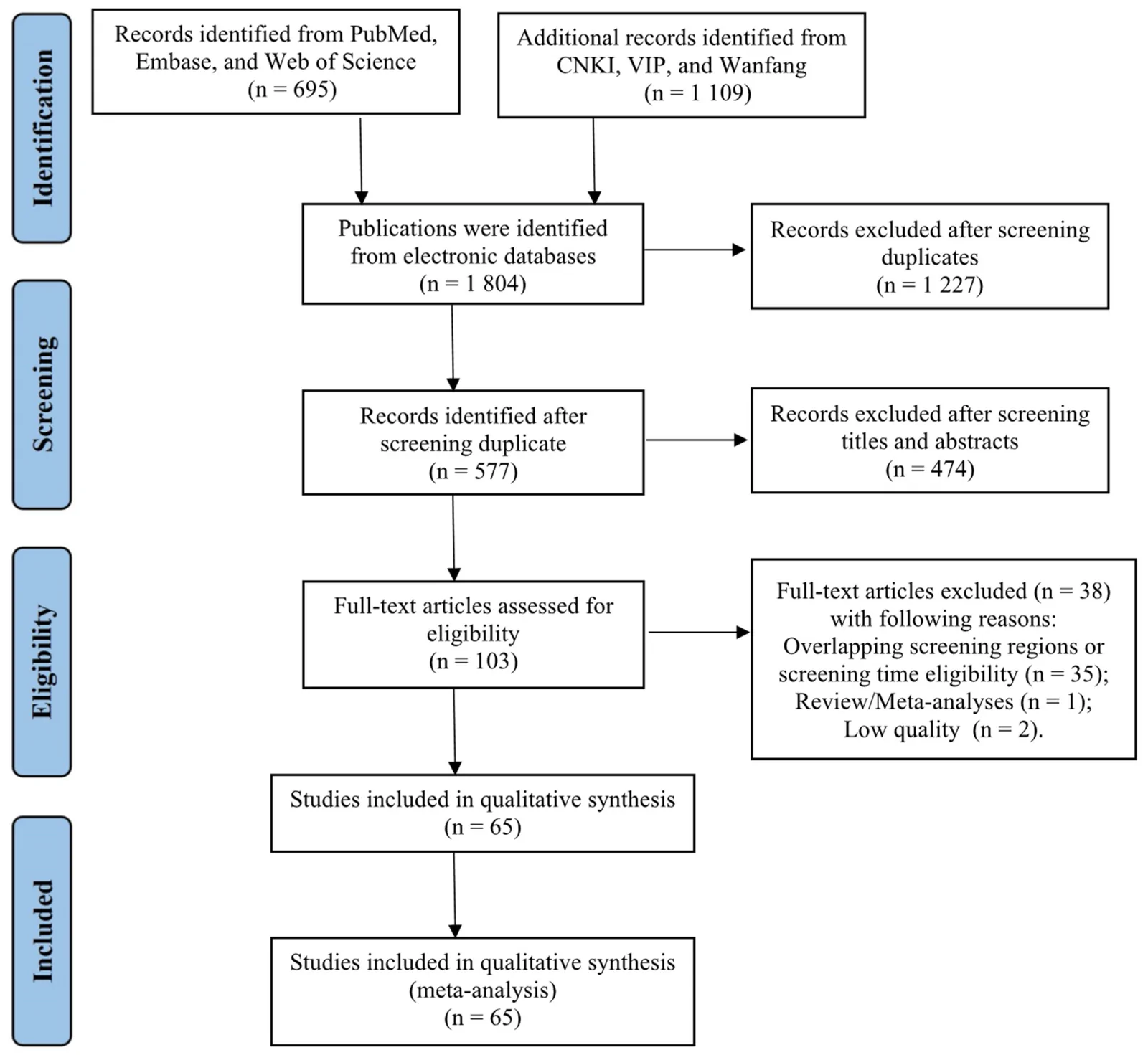
Flow chart of the study selection process.

A total of 1804 articles (695 in Chinese and 1109 in English) were retrieved from six databases in the initial search. After duplicate removal, 1227 articles were excluded, and the remaining records proceeded to the rescreening phase (title and abstract screening). During this phase, 474 articles were excluded for failing to meet the inclusion criteria, leaving 103 articles to enter the final screening phase (full-text review). After full-text assessment, an additional 38 articles were ultimately excluded, with the reasons for exclusion as follows: overlapping screening regions or time periods (35 articles), ineligible study design (1 article was a meta-analysis), and low methodological quality (2 articles). In summary, a total of 65 articles were finally included in this study for the subsequent meta-analysis. The flow diagram of the literature search and screening process is presented in Figure 1.

### 3.2. Study Characteristics

**Figure 2:**
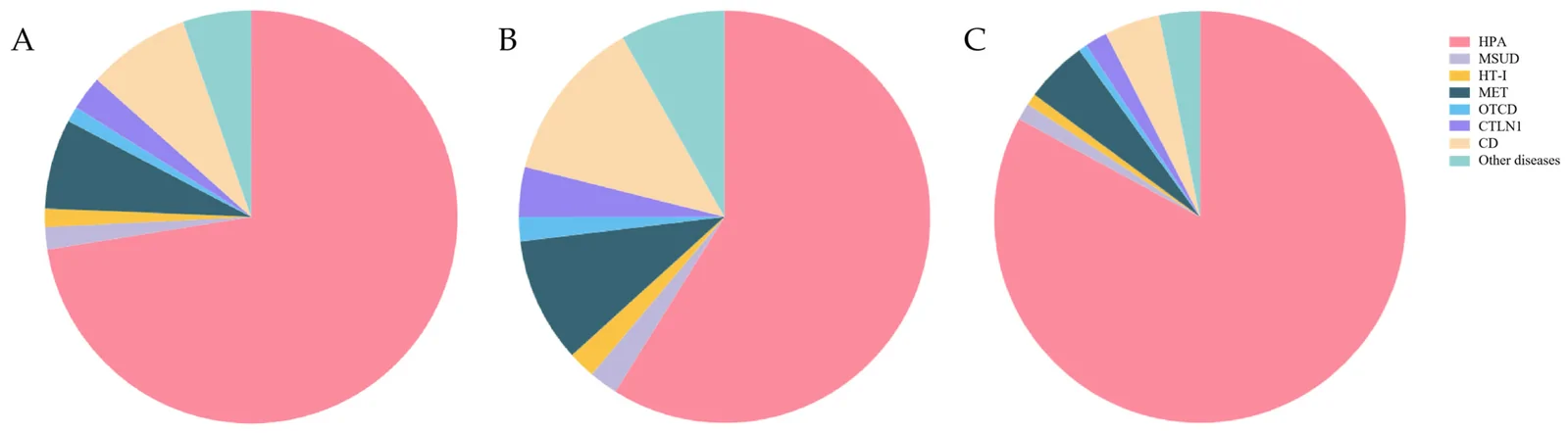
The disease spectrum of AAMs in Chinese newborns. (**A**) All regions. (**B**) Southern region. (**C**) Northern region.

The 65 included studies covered a total of 16,757,850 newborns, among whom 2928 cases were diagnosed with AAMs (Table 1). Of note, 67.99% (11,393,586/16,757,850) of the screened newborns were from southern China. Among the 2928 patients with AAMs, the seven most common diseases accounted for 94.64% of the total cases, including: hyperphenylalaninemia (HPA; 72.47%, 2122/2928), maple syrup urine disease (MSUD; 1.74%, 51/2928), tyrosinemia (HT; 1.43%, 42/2928), hypermethioninemia (MET; 7.04%, 206/2928), ornithine transcarbamylase deficiency (OTCD; 1.20%, 35/2928), citrullinemia type 1 (CTLN1; 2.70%, 79/2928), and citrin deficiency (CD; 8.06%, 236/2928; Figure 2A). Analysis of the included studies revealed significant geographical differences in the disease spectrum of AAMs across China (Figure 2B,C). In northern China, HPA was the absolutely predominant subtype of AAMs, accounting for 82.89% (1371/1654) of all cases in the region. Among the 725 HPA patients with available subtype data, the vast majority (94.48%, 685/725) were caused by phenylalanine hydroxylase deficiency. In contrast, the disease distribution in southern China was more diverse. Although HPA remained the leading subtype, its proportion (58.90%, 751/1275) was significantly lower than that in northern China. The proportion of phenylalanine hydroxylase deficiency in HPA cases from southern China (86.40%, 305/353) was lower than that in northern China, while the proportion of tetrahydrobiopterin deficiency was relatively higher.

### 3.3. The Result of Quality Assessment

Study methodological quality was scored using the 11-item AHRQ checklist, with scores ranging from 4 to 11 across the 65 eligible studies (mean score = 7.62). Stratified grading revealed 29 high-quality studies (8–11 points, 44.6% of total) and 36 moderate-quality studies (4–7 points, 55.4%); no low-quality studies (0–3 points) were retained after full-text screening. The most consistently satisfied AHRQ items included a clear definition of study population, standardized NBS protocols, and complete reporting of case and total screened newborn numbers. Minor reporting deficiencies were concentrated in three checklist domains: (1) incomplete statement of lost-to-follow-up screening infants; (2) insufficient description of diagnostic recheck workflows for borderline MS/MS results; (3) absent reporting of ethnicity stratification for regional newborn cohorts. Sensitivity stratification by study quality (high vs. moderate) was additionally performed in supplementary analyses, which demonstrated no material alteration to pooled incidence or north–south subgroup disparities, confirming that minor reporting gaps did not bias core meta-analytic outcomes.

### 3.4. Meta-Analysis Results

#### 3.4.1. AAM Incidence

**Figure 3:**
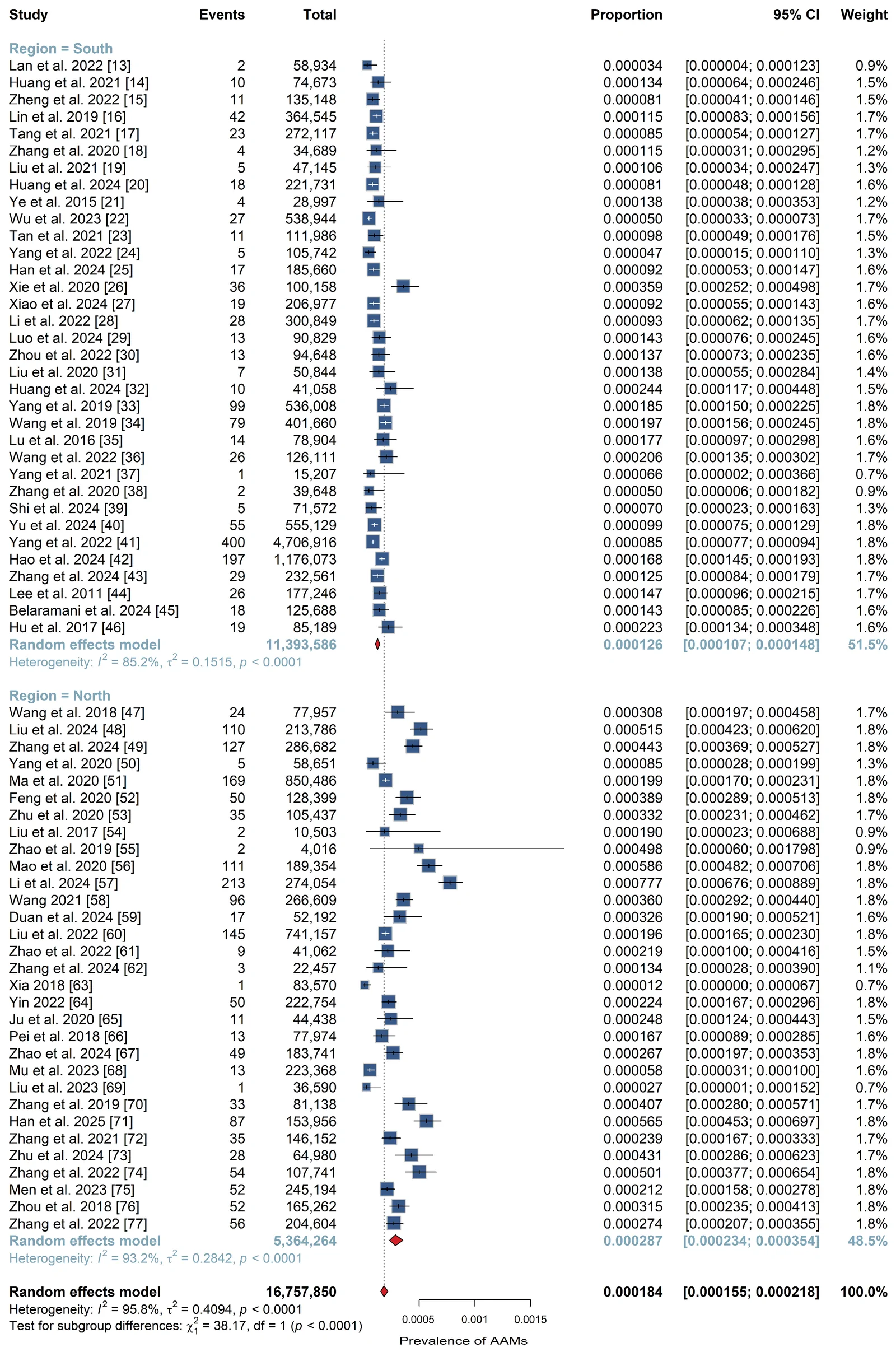
Meta-analysis of the incidence of AAMs between southern and northern China [13,14,15,16,17,18,19,20,21,22,23,24,25,26,27,28,29,30,31,32,33,34,35,36,37,38,39,40,41,42,43,44,45,46,47,48,49,50,51,52,53,54,55,56,57,58,59,60,61,62,63,64,65,66,67,68,69,70,71,72,73,74,75,76,77]. Subgroup meta-analysis of AAMs prevalence stratified by southern and northern regions of China. Blue squares represent the prevalence of individual studies; red diamonds indicate the pooled prevalence calculated by random-effects model. Horizontal solid lines correspond to the 95% confidence intervals (CIs) of each study. The vertical dashed line is the null reference line (prevalence = 0.001). Light blue squares denote studies from South China, dark blue squares represent studies from North China. The percentage in the “Weight” column shows the weight of each study in the meta-analysis. Heterogeneity indices (*I*^2^, *τ*^2^, *p*) and subgroup difference test results are presented at the bottom.

All included studies reported the incidence of AAMs. Owing to the high between-study heterogeneity *(I*^2^ = 95.8%, *p* < 0.0001), a random-effects model was applied in this study to analyze the incidence of AAMs in Chinese newborns. The meta-analysis showed that the pooled incidence of AAMs in Chinese newborns was 184.0 per million (95% CI 155.0–218.0 per million, Figure 3).

**Figure 4:**
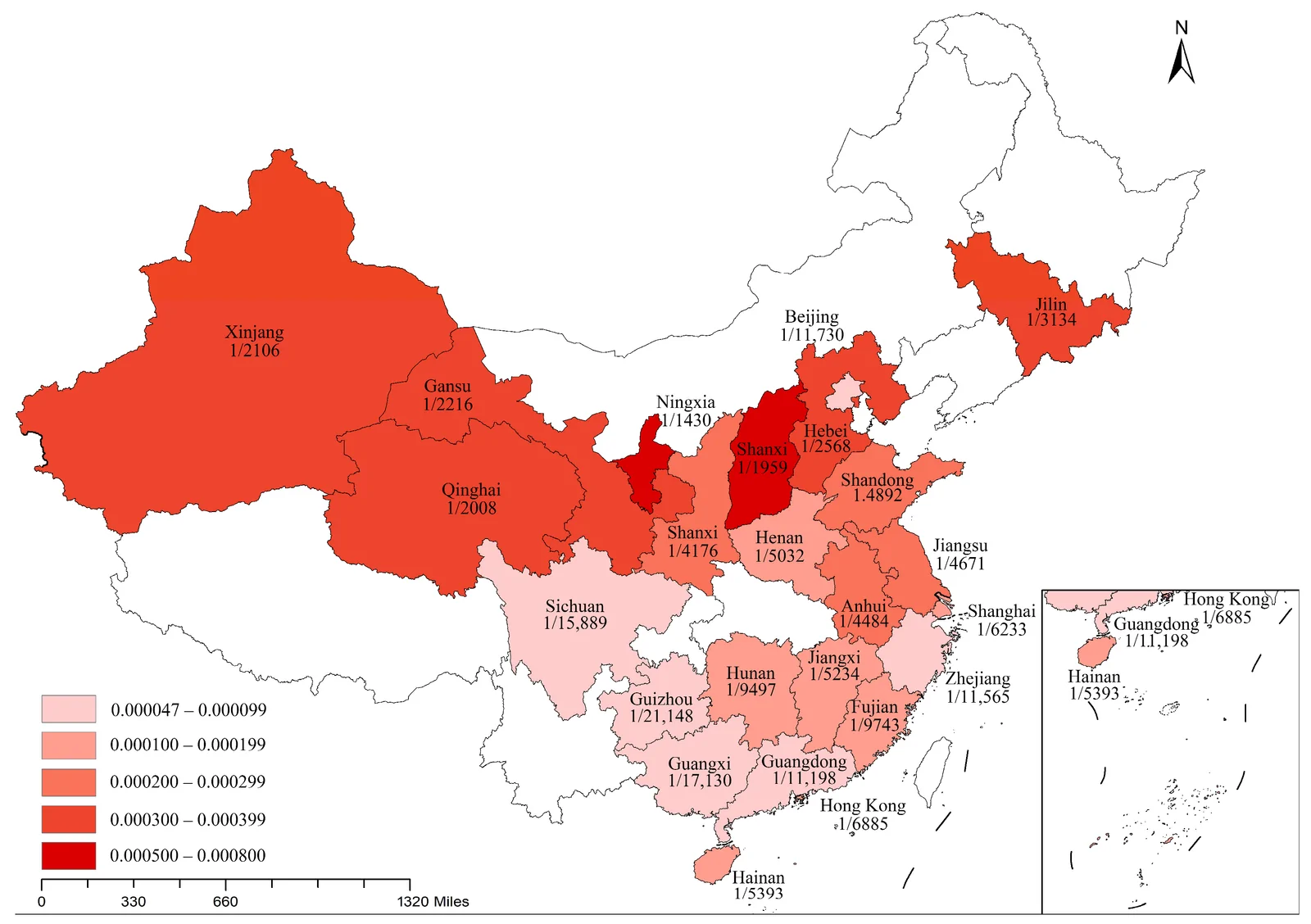
The schematic diagram shows the incidence of AAMs in different provinces of China.

Further regional subgroup analysis revealed that the incidence of AAMs in northern China (287.0 per million, 95% CI 234.0–354.0 per million) was significantly higher than that in southern China (126.0 per million, 95% CI 107.0–148.0 per million), and the difference between subgroups was statistically significant (*χ*^2^ = 38.17, *df* = 1, *p* < 0.0001, Figure 3 and Figure 4).

Substantial between-study heterogeneity was observed in the overall pooled analysis. Subgroup stratification by geographic region partially explained the observed heterogeneity, as the north–south disparity in incidence reached statistical significance. However, high heterogeneity remained within both the southern (*I*^2^ = 85.2%, *p* < 0.0001) and northern subgroups (*I*^2^ = 93.2%, *p* < 0.0001), indicating that geographic distribution was only one of the contributing factors. Other potential sources of heterogeneity include the 24-year time span of included studies, variations in MS/MS screening protocols and cut-off values across regions, differences in confirmatory diagnostic workflows, and wide variation in sample sizes among studies.

#### 3.4.2. Incidence of AAM Disease Spectrum

In this study, we performed a meta-analysis of the seven most common AAMs, namely HPA, MET, CD, CTLN1, MSUD, OTCD, and HT. Given the high between-study heterogeneity observed in studies on HPA (*I*^2^ = 96.0%, *p* < 0.0001), a random-effects model was used for the pooled analysis of its incidence. For the remaining six diseases, the between-study heterogeneity ranged from none to moderate (*I*^2^ ≤ 62.3%), and a random-effects model was also applied to generate pooled estimates. The results of the meta-analysis showed that the pooled incidence (per million) of HPA, MET, CD, CTLN1, MSUD, OTCD, and HT was 126.00 (95% CI 101.00–159.00), 18.00 (95% CI 14.00–23.00), 21.00 (95% CI 17.00–25.00), 10.00 (95% CI 8.00–13.00), 3.00 (95% CI 2.00–4.00), 2.00 (95% CI 1.00–3.00), and 7.00 (95% CI 6.00–10.00), respectively.

**Figure 5:**
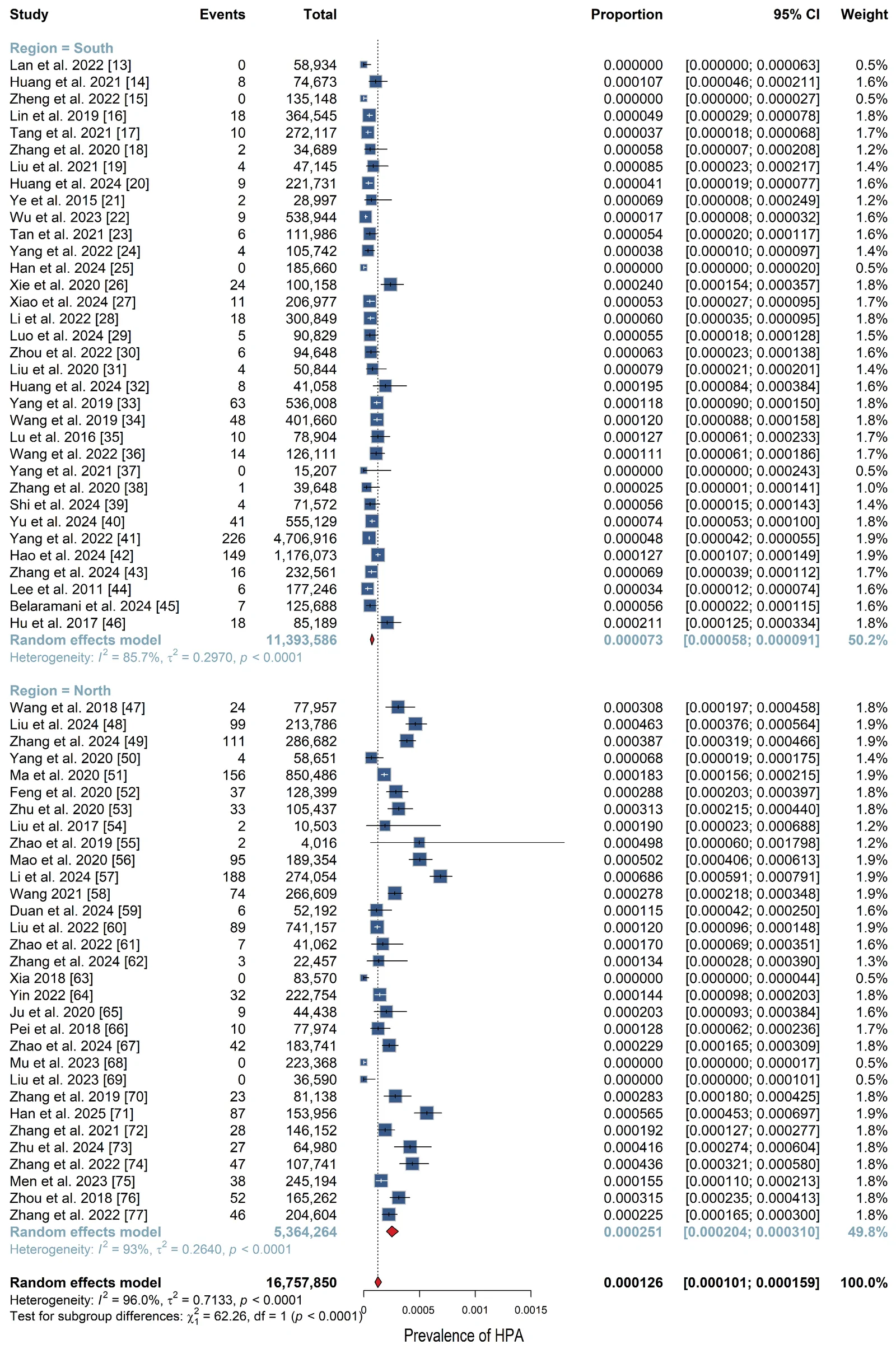
Meta-analysis of the incidence of HPA between southern and northern China [13,14,15,16,17,18,19,20,21,22,23,24,25,26,27,28,29,30,31,32,33,34,35,36,37,38,39,40,41,42,43,44,45,46,47,48,49,50,51,52,53,54,55,56,57,58,59,60,61,62,63,64,65,66,67,68,69,70,71,72,73,74,75,76,77].

Subgroup analysis showed that the incidence of HPA in northern China (251.00 per million, 95% CI 204.00–310.00) was significantly higher than that in southern China (73.00 per million, 95% CI 58.00–91.00), with a statistically significant difference between subgroups (*χ*^2^ = 62.26, *df* = 1, *p* < 0.0001; Figure 5). In contrast, no statistically significant difference was observed in the incidence of the other six diseases (MET, CD, CTLN1, MSUD, OTCD, and HT) between northern and southern China divided by the Qinling–Huaihe Line (all *p*–values > 0.05; Figures S1–S6).

**Figure 6:**
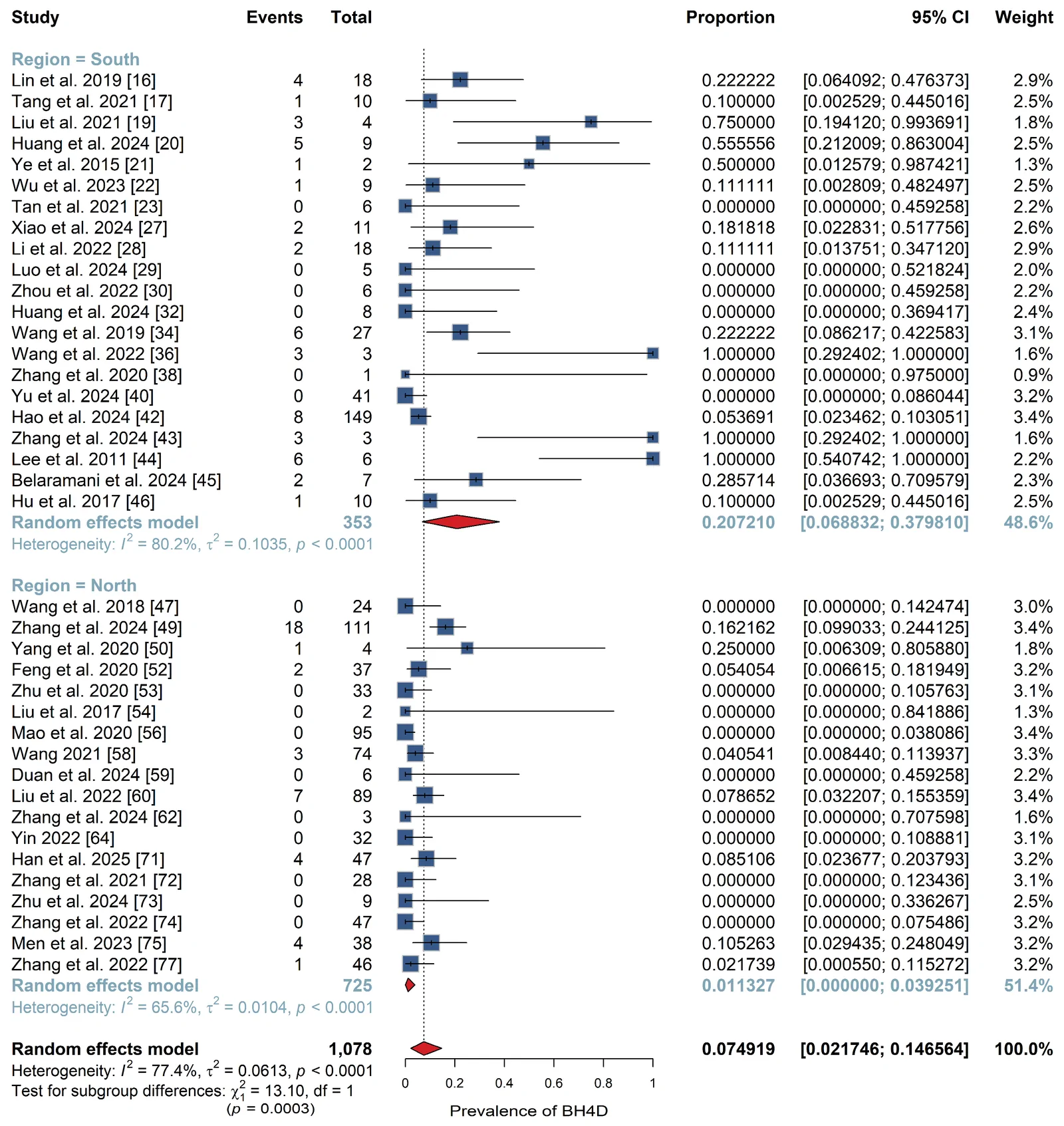
Meta-analysis of the incidence of BH4D between southern and northern China [16,17,19,20,21,22,23,27,28,29,30,32,34,36,38,40,42,43,44,45,46,47,49,50,52,53,54,56,58,59,60,62,64,71,72,73,74,75,77].

This study evaluated the constituent ratio of BH4D among all patients with HPA (including BH4D and phenylalanine hydroxylase deficiency) through a meta-analysis. A total of 39 studies were included, of which 21 were from southern China and 18 were from northern China. Owing to the high between-study heterogeneity (*I*^2^ = 77.4%, *p* < 0.0001), a random-effects model was adopted for pooled analysis. The overall pooled constituent ratio of BH4D among Chinese patients with HPA was 0.07 (95% CI 0.02–0.15). Subgroup analysis showed that the constituent ratio of BH4D was significantly higher in southern China (0.21, 95% CI 0.07–0.38) than in northern China (0.01, 95% CI 0.00–0.04) (χ^2^ = 13.10, *df* = 1, *p* = 0.0003; Figure 6). Among the included studies, 23 reported BH4D cases, comprising a total of 88 patients. Molecular genetic information was available for 78 patients from 17 studies. Of these, 76 patients harbored pathogenic variants in the PTS gene, consistent with 6-pyruvoyl-tetrahydropterin synthase (PTPS) deficiency, while 2 patients carried pathogenic variants in the QDPR gene, consistent with dihydropteridine reductase (DHPR) deficiency. The remaining 10 cases lacked reported molecular genetic results and therefore could not be further classified by specific enzyme deficiency.

#### 3.4.3. Publication Bias

Publication bias was assessed using funnel plots, Egger’s regression test, Begg’s rank correlation test, and trim-and-fill analysis. For overall AAMs, the funnel plot showed marked asymmetry. Egger’s test was statistically significant (*p* = 0.0267), whereas Begg’s test was not (*p* = 0.9278). The trim-and-fill method estimated 19 potentially missing studies and adjusted the pooled incidence from 184.0 (95% CI: 155.0–218.0) to 121.8 (95% CI: 90.7–157.4) per million, suggesting that the pooled estimate may have been influenced by publication bias.

Among the seven major subtypes, the degree of publication bias varied. For HPA, Egger’s test was significant (*p* = 0.0267), and trim-and-fill estimated 16 potentially missing studies, reducing the pooled incidence from 126.0 to 74.6 per million. For MET and CD, no statistically significant publication bias was detected (Egger’s *p* = 0.2124 and 0.3958, respectively), and the adjusted incidences changed only slightly, from 10.6 to 9.0 per million for MET and from 12.4 to 11.2 per million for CD, suggesting little influence of publication bias on the pooled estimates. For CTLN1, both Egger’s (*p* = 0.025) and Begg’s (*p* < 0.001) tests indicated funnel plot asymmetry. Trim-and-fill estimated nine potentially missing studies and reduced the pooled incidence from 3.5 to 2.6 per million. For MSUD, Egger’s test showed borderline significance (*p* = 0.0514), whereas Begg’s test was significant (*p* < 0.001). Trim-and-fill estimated 16 potentially missing studies and reduced the pooled incidence from 2.0 to 1.3 per million. For the rare subtypes OTCD and HT, the results of Egger’s and Begg’s tests were inconsistent, but trim-and-fill estimated 29 and 28 potentially missing studies, respectively, resulting in substantial reductions in the pooled incidences (OTCD: 1.4 to 0.1 per million; HT: 2.2 to 0.3 per million). For the proportion of BH4D among HPA cases, both Egger’s (*p* = 0.0064) and Begg’s (*p* = 0.0053) tests were statistically significant. Trim-and-fill estimated nine potentially missing studies and reduced the pooled constituent ratio from 10.90% to 4.14%.

Because several rare subtypes (particularly CTLN1, MSUD, OTCD, and HT) had extremely low event counts, the statistical power of publication bias tests was inherently limited. Therefore, the results for these rare disorders should be considered exploratory and interpreted with caution. Despite these adjustments, the north–south disparities for overall AAMs and HPA remained statistically significant, indicating that the observed regional pattern was robust to publication bias. Overall, the trim-and-fill analyses suggest that publication bias may have affected the absolute pooled incidence estimates, particularly for rare subtypes, whereas the principal epidemiological conclusions of this study remained unchanged.

## 4. Discussion

In this large-scale meta-analysis including more than 16 million newborns across 34 provincial-level regions in China, we provide a comprehensive synthesis of the epidemiology of AAMs detected through NBS. To our knowledge, this represents the largest national-level evidence base to date, systematically characterizing both overall incidence and geographic variation across China.

The pooled incidence of AAMs was estimated at 184.0 per million newborns (1 in 5435), placing China within the intermediate range globally. Compared with major regions worldwide, reported incidences vary substantially, including approximately 1 in 12,658 in the United States [78], 1 in 3745 in the United Kingdom [79], 1 in 3802 in Germany [79], 1 in 2988 in Italy [80], and 1 in 4418 in Russia [81]. Among East Asian countries, the estimated incidence of inherited AAMs is 1 in 40,000 in Japan [82] and 1 in 6090 in South Korea [83]. These differences likely reflect variation in genetic background, screening strategies, and disease inclusion criteria, limiting direct cross-country comparability. Overall, our findings indicate that China exhibits both shared and population-specific epidemiological features of AAMs.

A national consistency analysis showed that our estimates are broadly aligned with a large-scale Chinese screening study reporting an incidence of approximately 1 in 5800. Notably, we identified a clear and consistent north–south gradient, with higher incidence in northern China than in southern regions. This pattern was primarily driven by hyperphenylalaninemia (HPA), whereas no significant geographic differences were observed for other major AAM subtypes. These findings suggest that regional variation in overall AAM incidence is largely attributable to differences in HPA distribution.

The observed north–south disparity is likely driven by a combination of biological and non-biological factors, and should be interpreted cautiously given the ecological nature of the analysis. Importantly, several potential confounders may have contributed to the observed regional differences. First, population structure varies across China, with differences in ethnic composition that may influence the distribution of pathogenic variants associated with AAMs. Prespecified subgroup analysis stratified by the Qinling–Huaihe Line confirmed regional disparity as a major source. The incidence in northern China was over twice that in southern China (287.0 vs. 126.0 per million, *p* < 0.0001), mainly driven by the uneven distribution of HPA. After stratification, within-group heterogeneity decreased moderately (south: *I*^2^ = 85.2%; north: *I*^2^ = 93.2%), indicating geographic factors partially explain the overall heterogeneity. Second, variability in healthcare access and newborn screening implementation, including differences in program coverage, timing of screening expansion, and follow-up systems, may lead to differential case ascertainment across regions. Third, differences in socioeconomic development and healthcare resources may further affect screening uptake, diagnostic confirmation, and reporting completeness. In addition, genetic variation in PAH-related pathways may also partially contribute to the higher incidence of HPA observed in northern China; however, this biological explanation should be considered alongside these potential systematic sources of bias. Furthermore, differences in study design, screening coverage, case ascertainment, and confirmatory diagnostic criteria among provinces may have contributed to the residual heterogeneity, which could not be fully adjusted for using aggregate-level data in this meta-analysis. Future studies with individual-level data and standardized nationwide screening protocols are required to disentangle the relative contributions of these factors.

From a clinical and public health perspective, these findings support regionally tailored NBS strategies in China. In northern regions, where HPA constitutes the dominant disease burden, optimized phenylalanine-based screening and rapid confirmatory pathways may improve efficiency. In southern regions, a relatively higher proportion of citrin deficiency and tetrahydrobiopterin deficiency highlights the importance of enhanced differential diagnosis and expanded molecular testing in selected cases.

Several limitations should be acknowledged. First, substantial heterogeneity was observed across the included studies. As the *I*^2^ statistic reflects true between-study variability rather than sampling error, such heterogeneity is common in observational meta-analyses [84]. In our study, it likely resulted from differences in screening methodologies, population characteristics, and regional implementation of newborn screening [85,86]. A random-effects model was therefore applied, and sensitivity analyses confirmed the robustness of the pooled estimates. Nevertheless, heterogeneity remains an inherent limitation, and the pooled incidence should be interpreted with appropriate caution. Second, potential overlap between study populations could not be fully excluded due to limited reporting granularity, although conservative exclusion criteria were applied. Third, publication bias may have influenced the absolute incidence estimates. The funnel plot asymmetry and trim-and-fill analysis suggested that studies with smaller effects or lower incidence estimates may have been underrepresented, potentially resulting in an overestimation of the pooled incidence. Nevertheless, the adjusted estimates remained within the same order of magnitude, and the principal conclusions regarding the regional distribution and disease spectrum of AAMs were unchanged. Therefore, publication bias is more likely to affect the magnitude of absolute incidence estimates rather than the overall epidemiological pattern identified in this study. Finally, the ecological design and lack of individual-level data limit adjustment for potential confounders such as ethnicity and healthcare access.

In conclusion, this meta-analysis provides robust national evidence on the incidence and geographic distribution of AAMs in China, demonstrating an apparent north–south gradient in overall AAM incidence, which was largely attributable to the marked regional difference in HPA. These findings highlight the need for regionally optimized NBS strategies and support the development of more precise and standardized national screening frameworks.

## 5. Conclusions

This study obtained a robust pooled incidence estimate of AAMs among Chinese newborns screened via tandem mass spectrometry (MS/MS), at 184.0 per million live births (95% CI 155.0–218.0 per million, equivalent to 18.4 per 100,000), and revealed significant north–south regional disparities. The overall incidence showed an apparent north–south difference, which was largely attributable to the substantially higher incidence of HPA in northern China rather than consistent differences across all AAM subtypes. The disease spectrum composition showed regional variations, with a higher diversity of disorders identified in southern China and a significantly higher proportion of tetrahydrobiopterin deficiency among HPA cases. These findings suggest that AAM epidemiology in China exhibits regional characteristics, although the observed geographic differences should be interpreted cautiously, considering the dominant contribution of HPA. This study provides important epidemiological evidence for optimizing NBS strategies and the rational allocation of regional medical resources.

## Figures and Tables

**Table 1:** Characteristics of studies included in the meta-analysis.

South or North of China	Reference (Author/Year)	Area		Years	Screening Cases	AAMs Cases	Incidence	Disease Spectrum of AAMs										AHRQ Scores
		Province	City					HPA	PKU	BH4D	MSUD	HT	MET	OTCD	CTLN	CD	Other Disease	
South	Lan et al. 2022 [13]	Fujian	Longyan	2018–2020	58,934	2	1/5358						1				1	9
	Huang et al. 2021 [14]	Fujian	Nanping	2016–2019	74,673	10	1/2987	8					1				1	8
	Zheng et al. 2022 [15]	Fujian	Ningde	2016–2020	135,148	11	1/2816						1		4	5	1	7
	Lin et al. 2019 [16]	Fujian	Quanzhou	2014–2018	364,545	42	1/2804		14	4			3	2	2	10	7	9
	Tang et al. 2021 [17]	Guangdong	Guangzhou	2015–2020	272,117	23	1/3444		9	1	1		2		2	6	2	8
	Zhang et al. 2020 [18]	Guangdong	Huizhou	2017–2019	34,689	4	1/3469	2							1		1	8
	Liu et al. 2021 [19]	Guangdong	Meizhou	2019–2021	47,145	5	1/4286		1	3						1		7
	Huang et al. 2024 [20]	Guangdong	Zhongshan	2014–2023	221,731	18	1/5993		4	5				2		7		7
	Ye et al. 2015 [21]	Guangdong	Dongguan	2012–2015	28,997	4	1/4833		1	1	1	1						9
	Wu et al. 2023 [22]	Guangxi		2011–2020	538,944	27	1/19,961		8	1			1	2	4	11		7
	Tan et al. 2021 [23]	Guangxi	Liuzhou	2012–2020	111,986	11	1/3733		6			1		2			2	7
	Yang et al. 2022 [24]	Guizhou	Guiyang	2015–2019	105,742	5	1/7553	4				1						7
	Han et al. 2024 [25]	Hainan	Haikou	2017–2021	185,660	17	1/2110				1	1	3			8	4	9
	Xie et al. 2020 [26]	Hainan		2014–2019	100,158	36	1/1890	24					8		2		2	7
	Xiao et al. 2024 [27]	Hunan	Huaihua	2015–2021	206,977	19	1/3000		9	2	1		1			6		8
	Li et al. 2022 [28]	Hunan	Changsha	2016–2020	300,849	28	1/4237		16	2		1	2		2	5		9
	Luo et al. 2024 [29]	Hunan	Zhuzhou	2019–2022	90,829	13	1/3027		5				1	2		4	1	7
	Zhou et al. 2022 [30]	Hunan	Shaoyang	2016–2020	94,648	13	1/4115		6			2			1	4		7
	Liu et al. 2020 [31]	Jiangsu	Changzhou	2015–2018	50,844	7	1/2676	4					1		1	1		8
	Huang et al. 2024 [32]	Jiangsu	Jiangyin	2019–2022	41,058	10	1/2281		8				2					7
	Yang et al. 2019 [33]	Jiangsu	Nanjing, Yangzhou and Lianyungang	2014–2018	536,008	99	1/2763	63			2	4	13	1	2	3	11	7
	Wang et al. 2019 [34]	Jiangsu	Suzhou	2014–2018	401,660	79	1/3163	21	21	6	1	4	13	3	2		8	9
	Lu et al. 2016 [35]	Jiangsu	Yancheng	2014–2015	78,904	14	1/4384	10							1		3	7
	Wang et al. 2022 [36]	Jiangxi		2016–2021	126,111	26	1/2581	11		3		1	3	1		5	2	7
	Yang et al. 2021 [37]	Jiangxi	Shangrao	2018–2019	15,207	1	1/1857									1		8
	Zhang et al. 2020 [38]	Sichuan	Sichuan	2017–2018	39,648	2	1/2046		1							1		7
	Shi et al. 2024 [39]	Sichuan	Yibin	2019–2021	71,572	5	1/1940	4							1			9
	Yu et al. 2024 [40]	Zhejiang	Ningbo	2014–2023	555,129	55	1/3802		41			1	3		2	3	5	7
	Yang et al. 2022 [41]	Zhejiang		2009–2021	4,706,916	400	1/2643	226			14	5	38	6	18	60	33	7
	Hao et al. 2024 [42]	Shanghai		2003–2022	1,176,073	197	1/2468		141	8	6	2	20		4	8	8	7
	Zhang et al. 2024 [43]	Shanghai		2010–2020	232,561	29	1/4303	13		3	1		7	1		3	1	7
	Lee et al. 2011 [44]	Hong Kong		2005–2009	177,246	26	1/4535			6		3		2	1	12	2	8
	Belaramani et al. 2024 [45]	Hong Kong		2015–2022	125,688	18	1/3000		5	2	1						10	9
	Hu et al. 2017 [46]	Anhui	Hefei	2015–2016	85,189	19	1/4153	8	9	1			1					7
North	Wang et al. 2018 [47]	Gansu		2015–2016	77,957	24	1/4122		24									7
	Liu et al. 2024 [48]	Gansu		2021–2023	213,786	110	1/2674	99			2	1		2	5		1	7
	Zhang et al. 2024 [49]	Gansu		2018–2021	286,682	127	1/2840		93	18	3	1		3	2	6	1	7
	Yang et al. 2020 [50]	Beijing		2014–2019	58,651	5	1/2688		3	1						1		7
	Ma et al. 2020 [51]	Henan		2013–2019	850,486	169	1/1474	156			1		1			4	7	9
	Feng et al. 2020 [52]	Hebei	Shijiazhuang	2014–2018	128,399	50	1/1593		35	2	1	2	5			2	3	7
	Zhu et al. 2020 [53]	Jilin		2015–2018	105,437	35	1/3666		33								2	8
	Liu et al. 2017 [54]	Jilin	Liaoyuan	2011–2016	10,503	2	1/2085		2									7
	Zhao et al. 2019 [55]	Qinghai		2018–2018	4016	2	1/1259	2										8
	Mao et al. 2020 [56]	Ningxia		2016–2019	189,354	111	1/2452		95		3		6		1		6	9
	Li et al. 2024 [57]	Ningxia		2017–2021	274,054	213	1/3051	188			4	2	7		6	4	2	7
	Wang 2021 [58]	Shandong	Heze	2015–2020	266,609	96	1/1339		71	3	1	3	5			11	2	7
	Duan et al. 2024 [59]	Shandong	Jining	2022	52,192	17	1/1183		6				5		3	3		9
	Liu et al. 2022 [60]	Shandong	Jining	2014–2021	741,157	145	1/982		82	7	4	2	23	3	6	15	3	7
	Zhao et al. 2022 [61]	Shandong	Langfang	2020	41,062	9	1/1130	7					2					8
	Zhang et al. 2024 [62]	Shandong	Liaocheng	2020–2021	22,457	3	1/1631		3									7
	Xia 2018 [63]	Shandong	Linyi	2014–2017	83,570	1	1/5111						1					8
	Yin 2022 [64]	Shandong	Taian	2013–2019	222,754	50	1/2281		32				2		3	3	10	7
	Ju et al. 2020 [65]	Shandong	Weihai	2018–2019	44,438	11	1/2807	9						1		1		7
	Pei et al. 2018 [66]	Shandong	Weifang	2013–2016	77,974	13	1/5223	10									3	9
	Zhao et al. 2024 [67]	Shandong	Zaozhuang	2015–2023	183,741	49	1/2273	42					4			3		7
	Mu et al. 2023 [68]	Shandong	Zibo	2015–2022	223,368	13	1/2116				1	1	4			4	3	7
	Liu et al. 2023 [69]	Shandong	Rizhao	2016–2022	36,590	1	1/2437									1		8
	Zhang et al. 2019 [70]	Shanxi	Taiyuan	2016–2018	81,138	33	1/2517	23					7		2		1	7
	Han et al. 2025 [71]	Shanxi	Changzhi	2015–2020	153,956	87	1/2978	40	43	4								7
	Zhang et al. 2021 [72]	Shanxi	Xian	2014–2019	146,152	35	1/3539		28			1		1		4	1	8
	Zhu et al. 2024 [73]	Xinjiang	Wulumuqi	2018–2022	64,980	28	1/1656	18	9			1						8
	Zhang et al. 2022 [74]	Xinjiang		2019–2024	107,741	54	1/1193		47		1		6					7
	Men et al. 2023 [75]	Jiangsu	Lianyungang	2015–2021	245,194	52	1/1949		34	4	1					7	6	9
	Zhou et al. 2018 [76]	Jiangsu	Xuzhou	2015–2017	165,262	52	1/1547	52										7
	Zhang et al. 2022 [77]	Jiangsu	Suqian	2016–2021	204,604	56	1/1476		45	1		1	3	1	1	3	1	7

## Data Availability

All data generated or analyzed during this study are included in the article; further inquiries can be directed to the corresponding author.

## Supplementary Materials

The following supporting information can be downloaded at: https://www.mdpi.com/article/10.3390/ijns12030061/s1: Figure S1: Meta-analysis of the incidence of MET between southern and northern China; Figure S2: Meta-analysis of the incidence of CD between southern and northern China; Figure S3: Meta-analysis of the incidence of CTLN1 between southern and northern China; Figure S4: Meta-analysis of the incidence of MSUD between southern and northern China; Figure S5: Meta-analysis of the incidence of OTCD between southern and northern China; Figure S6: Meta-analysis of the incidence of HT between southern and northern China; Table S1: Agency for Healthcare Research and Quality (AHRQ) methodology checklist; Attachment S1: Detailed Search Strategies.

## Abbreviations

The following abbreviations are used in this manuscript:

AAMs	Amino acid metabolism disorders
NBS	Newborn screening
MS/MS	Tandem mass spectrometry
HPA	Hyperphenylalaninemia
MET	Hypermethioninemia
CD	Citrin deficiency
CTLN1	Citrullinemia type 1
MSUD	Maple syrup urine disease
OTCD	Ornithine transcarbamylase deficiency
HT	Tyrosinemia
BH4D	Tetrahydrobiopterin Deficiency
