# Supplementary material for: A Comprehensive Meta-Analytical Investigation into the Incidence of Neonatal Amino Acid Metabolic Disorders Across China
Source: Int J Neonatal Screen. 2026 Jul 30;12(3):61. doi: 10.3390/ijns12030061 (PMC13510947; doi:10.3390/ijns12030061)
Supplement: Supplementary file 1 [file IJNS-12-00061-s001.zip › Attachment S1.Detailed Search Strategies_.pdf]

Attachment S1: Detailed Search Strategies

Databases Searched: PubMed, Embase (Ovid), Web of Science Core Collection, China National Knowledge Infrastructure (CNKI), Wanfang Database, and VIP (Chongqing VIP) Chinese Science and Technology Periodical Database.

Limits: Humans, English & Chinese language.

1. PubMed (National Library of Medicine)

Search string:

(("amino acid metabolism, inborn errors"[Mesh] OR "amino acid disorders"[Title/Abstract] OR "amino acid metabolic disorders"[Title/Abstract] OR "inborn errors of metabolism"[Titl e/Abstract]) AND ("newborn screening"[Mesh] OR "neonatal screening"[Title/Abstract] OR "newborn screening"[Title/Abstract]) AND ("China"[Mesh] OR "Chinese"[Title/Abstract] OR "China"[Title/Abstract]))AND ("2002/01/01"[Date - Publication] : "2025/12/31"[Date - Publ ication])

2. Embase (Ovid)

Search string:

(exp amino acid blood level/ or exp amino acid metabolism/ or (amino acid disorder* or amino acid metabolic disorder* or inborn error* of metabolism).ti,ab,kw.)AND (newborn sc reening/ or (newborn screening or neonatal screening).ti,ab,kw.)AND (exp China/ or (China or Chinese).ti,ab.)AND [2002-2025]/py

3. Web of Science Core Collection

Search string:

TS=("amino acid disorders" OR "amino acid metabolic disorders" OR "inborn errors of me tabolism") AND TS=("newborn screening" OR "neonatal screening") AND TS=(China OR Chinese)

4. China National Knowledge Infrastructure (CNKI)

Search string :

(Topic='Amino acid metabolic disease' OR Topic='Amino acid metabolic disorder' OR Topi c='Inherited metabolic disease') AND (Topic='Newborn screening' OR Topic='Neonatal dise ase screening')

Date range: 2002-2025.

5. Wanfang Database

Search string:

(Topic:(Amino acid metabolic disease) OR Topic:(Inherited metabolic disease) OR Topic:(A mino acid metabolic disorder)) AND (Topic:(Newborn screening))

Time span: 2002-2025.

6. VIP (Chongqing VIP) Chinese Science and Technology Periodical Database

Search string:

(Title or Keywords='Amino acid metabolic disease' OR Title or Keywords='Inherited metabolic disease' OR Title or Keywords='Amino acid metabolic disorder') AND (Title or Keywords='Newborn screening')

A total of 1,768 records were retrieved from all electronic databases before duplicate removal, including 659 records from English databases and 1,109 records from Chinese databases.
