# Supplementary material for: A Comprehensive Meta-Analytical Investigation into the Incidence of Neonatal Amino Acid Metabolic Disorders Across China
Source: Int J Neonatal Screen. 2026 Jul 30;12(3):61. doi: 10.3390/ijns12030061 (PMC13510947; doi:10.3390/ijns12030061)
Supplement: Supplementary file 1 [file IJNS-12-00061-s001.zip › Table S1.Agency for Healthcare Research and Quality (AHRQ) methodology checklist.pdf]

Table S1 Agency for Healthcare Research and Quality (AHRQ) methodology checklist

#	Item	Question	Yes	No	Unclear
1	Source of Information	Was the source of data (e.g., patients, medical records) clearly described and appropriate?			
2	Inclusion/Exclusion Criteria	Were the inclusion and exclusion criteria pre-specified and clearly stated?			
3	Time Frame	Was the period of patient recruitment or data collection specified?			
4	Representativeness	Was the study population representative of a defined community or source population?			
5	Sample Size Justification	Was there an explanation of how the sample size was determined (e.g., power calculation)?			
6	Assessment of Exposure	Was the exposure assessed in a valid and reliable way for all participants?			
7	Temporal Relationship	If comparing groups, was it clear that the outcome was not present at baseline?			
8	Different Levels of Exposure	If relevant, were different levels (doses) of the exposure assessed?			
9	Confounding	Were potential confounders identified and strategies to address them described (e.g., multivariate analysis)?			
10	Assessment of Outcome	Was the outcome assessed in a valid and reliable way for all participants?			
11	Follow-up & Attrition	Was follow-up time sufficient and loss-to-follow-up reported and accounted for?			
